# Spontaneous Coronary Artery Dissection as an Uncommon Case of Non-ST-Elevation Myocardial Infarction: A Case Report and Review

**DOI:** 10.7759/cureus.76080

**Published:** 2024-12-20

**Authors:** Carlos Garibay, Kristy Leker

**Affiliations:** 1 Internal Medicine, Eisenhower Medical Center, Rancho Mirage, USA

**Keywords:** non-st segment elevation myocardial infarction (nstemi), nstemi, scad, scad management, spontaneous coronary artery dissection, womens health

## Abstract

Spontaneous coronary artery dissection (SCAD) is a rare condition that frequently goes undiagnosed. Still, it is becoming an increasingly recognized cause of acute coronary syndrome (ACS), predominantly in middle-aged women with few or no cardiovascular risk factors. We present a case of a 53-year-old female with traditional cardiovascular risk factors, who presented with typical anginal symptoms and was diagnosed with SCAD in the mid to distal left anterior descending artery (LAD). Although she presented with typical risk factors not associated with SCAD, coronary angiography confirmed the diagnosis, and she was successfully managed conservatively with dual antiplatelet therapy (DAPT). This case represents the importance of SCAD remaining as a differential diagnosis even in patients with traditional cardiovascular risk factors. Furthermore, it emphasizes the importance of prompt recognition, appropriate imaging, and individualized management for patients presenting with SCAD, especially as current guidelines lack standardization for follow-up after conservative therapy. This underscores the need for further research to better understand SCAD’s etiology and potential advancements in treatment.

## Introduction

Spontaneous coronary artery dissection (SCAD) is a rare cause of acute coronary syndrome (ACS) accounting for approximately 4% of overall cases; however, this is likely an underestimate due to its often misdiagnosis [1]. Most patients are young to middle-aged women with few or no cardiovascular risk factors and typical known associations with SCAD are fibromuscular dysplasia, pregnancy, and stress [2]. SCAD is defined as an epicardial coronary artery injury not associated with trauma or atherosclerosis. The typical etiology is a spontaneous tear within the coronary artery wall, creating a false lumen that will obstruct blood flow causing an intramural hematoma (IMH) [3]. Coronary angiography remains the gold standard of diagnosis for SCAD and it is used to define the variations of SCAD presentation. When diagnosis is uncertain, intracoronary imaging with optical coherence tomography (OCT) or intravascular ultrasound (IVUS) can be utilized as a diagnostic tool [1]. Herein, we present a case of a 53-year-old female with SCAD that highlights the importance of maintaining a high level of suspicion for SCAD in young women, even with typical cardiovascular risk factors, to ensure prompt diagnosis and management.

## Case presentation

Our patient was a 53-year-old female with a medical history of type 2 diabetes mellitus (DM), essential hypertension (HTN), and hyperlipidemia (HLD) who presented with worsening chest discomfort.

The patient’s chronic conditions were diagnosed within the last five years and were well controlled with her home medications of metformin 500 mg twice a day, losartan 50 mg daily, and atorvastatin 40 mg daily. Her last hemoglobin A1c (HbA1c) was 6.7% six months prior to admission. She was a lifelong nonsmoker who drank alcohol socially, about one to two drinks a week. Her family history was significant for HTN and DM.

The patient was in her usual state of health until the evening prior, when she began to experience chest discomfort. Initially, she attributed the sensation to anxiety or muscle strain and continued with her normal activities. However, upon waking the next morning, the chest discomfort persisted and intensified, evolving into significant squeezing left-sided chest pain radiating to her jaw and left arm, accompanied by epigastric pain and nausea. She reported that she had never felt anything like this before, prompting her to seek care in the emergency room. She denied any previous cardiac events, as well as symptoms such as paroxysmal nocturnal dyspnea, orthopnea, lower extremity edema, dyspnea, palpitations, or syncope.

**Figure 1:**
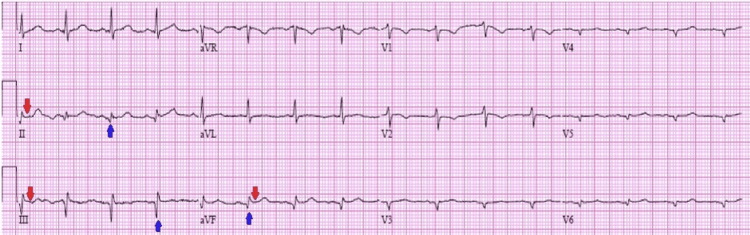
Initial electrocardiogram. The initial electrocardiogram (EKG) illustrates borderline inferior ST elevations in leads II, III, and augmented vector foot (aVF) (red arrows) with inferior Q waves (blue arrows). There is also poor R-wave progression seen, as the R-wave has an atypical decreased amplitude in V1-V6.

Upon presentation to the emergency room, her blood pressure was elevated to 153/83 mmHg, and the rest of her vital signs were within normal limits. She reported significant, non-reproducible chest pain. Her physical exam had no abnormal heart sounds and lung auscultation was clear, and no lower extremity edema or jugular venous distention was noted. Her initial laboratory tests were within normal limits with a non-fasting glucose level of 118 mg/dL (normal = 70-105 mg/dL) and B-type natriuretic peptide (BNP) was 89.0 pg/mL (normal = 1.0-100.0 pg/mL). Due to her presentation of chest pain, high-sensitivity troponin (normal = <14 pg/mL) was collected. Her first result was 1,374 pg/mL, which trended upwards to 1,389 pg/mL two hours later, and then 1,833 pg/mL four hours later. Initial electrocardiogram (EKG) revealed borderline inferior ST elevations in leads (II, III, and augmented vector foot (aVF)) with inferior Q waves with poor R-wave progression (Figure 1).

For a complete work-up of her chronic conditions and risk factors associated with ACS, her HbA1c and lipid panel were collected, which revealed HbA1c of 6.3% (normal = 4.0-6.0%), low-density lipoprotein (LDL) of 65 mg/dL (goal of less than 75 mg/dL with patients with diabetes), and cholesterol of 154 (normal = <200 mg/dL).

**Figure 2:**
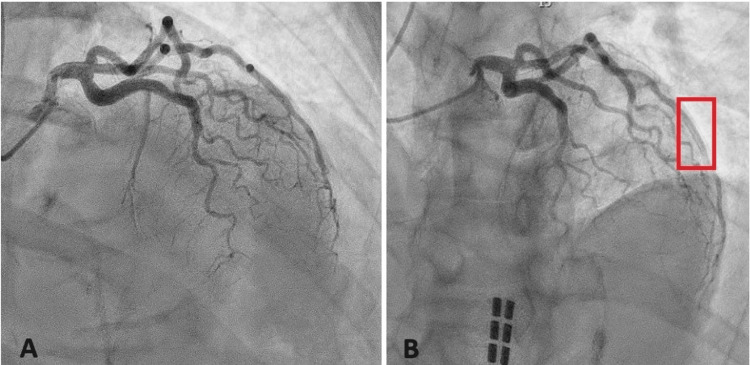
Coronary angiography. Coronary angiogram in the left anterior oblique (LAO) view showing spontaneous coronary artery dissection (SCAD) of the mid to distal left anterior descending artery (LAD). The progression of the dye is seen from panels A to B to illustrate the evidence of dye “hang-up” within the apical LAD consistent with SCAD (red box). Localized dye “hang-up” occurs when the contrast injected during cardiac catheterization inserts into the false lumen creating a dual lumen appearance.

Her electrocardiogram (EKG) changes and elevated troponin levels prompted her to receive aspirin 325 mg once, heparin 25,000 units in dextrose 5% 500 mL infusion, and sublingual nitroglycerin 0.4 mg in the emergency department. She continued to have ongoing chest discomfort and was started on a nitroglycerin infusion of 200 mcg/mL with a one-time morphine 4 mg injection. Cardiology was consulted from the emergency room and she was taken urgently for a coronary angiogram within 30 minutes of presentation. Cardiac catheterization revealed spontaneous coronary artery dissection (SCAD) involving the mid to distal left anterior descending artery (LAD) (Figure 2). Since SCAD was apparent on coronary angiography, intravascular imaging was not performed in her case.

No percutaneous coronary intervention (PCI) was indicated. The patient recovered from cardiac catheterization without issue. She underwent conservative management for SCAD with aspirin 81 mg indefinitely and clopidogrel 75 mg daily for one year. For her anginal symptoms, she was started on metoprolol tartrate 25 mg twice daily. Two days post-cardiac catheterization, her anginal symptoms resolved, and she was discharged on her fourth day of hospitalization.

Upon discharge, she would continue conservative therapy for SCAD along with metoprolol tartrate for her anginal symptoms. She was also instructed to continue with metformin 500 mg twice a day, losartan 50 mg daily, and atorvastatin 40 mg daily due to her adequate control of her chronic conditions and for prevention of coronary artery disease. She was recommended no strenuous exercise for a minimum of three to six months post-SCAD, to allow time for the artery to heal. Follow-up was arranged with her primary care provider and cardiologist within a week of discharge.

## Discussion

SCAD differs from typical ACS that occurs due to a plaque rupture; instead, it is due to a spontaneous tear in the coronary artery wall, creating a false lumen that can obstruct blood flow, typically with an IMH, as seen in this patient’s case. As discussed prior, the exact mechanisms behind SCAD remain unclear. The first theory is the "inside-out" theory, which suggests that an intimal tear allows blood from the true lumen to enter and form a false lumen. The second, or "outside-in" theory, proposes that a spontaneous hemorrhage originates from the vasa vasorum within the vessel wall, leading to dissection [1,3]. Further research is necessary to understand SCAD’s etiology, as this knowledge could assist in developing more specialized diagnostic algorithms and treatment plans in the future.

Both theories share the concept of an inciting event that contributes to SCAD formation. Those who experience SCAD are typically women in their fourth to fifth decade of life and the majority are reported within the Caucasian population [3]. Patients with SCAD report a higher frequency of precipitating events compared to those with other types of ACS, with extreme physical or emotional stress being the most commonly reported triggers [3]. Although SCAD predominantly affects women with conditions like fibromuscular dysplasia (FMD), this case demonstrates that it can also occur in women with traditional cardiovascular risk factors, such as hypertension and hyperlipidemia [4]. Additionally, SCAD is the leading cause of myocardial infarction (MI) in pregnant or postpartum patients and carries a poorer prognosis than SCAD cases unrelated to pregnancy. Unlike general SCAD, this subset’s common comorbidities include hypertension, hyperlipidemia, depression, migraines, advanced maternal age, and infertility treatment [3]. SCAD is an often underdiagnosed condition, especially in younger women, as it commonly presents in otherwise healthy individuals with symptoms resembling those of other ACS forms. Therefore, the differential diagnosis of SCAD is similar to that of ACS, including coronary vasospasm, microvascular ischemia, myocarditis, and Takotsubo cardiomyopathy.

Misdiagnosis of SCAD can lead to delayed or inappropriate treatment [5,6]. SCAD is diagnosed solely through coronary angiography and should be performed promptly [3]. Upon coronary angiography, most cases present with a narrowing that is secondary to IMH. When coronary angiography does not confirm the diagnosis, additional intracoronary imaging with OCT or IVUS can be utilized, although intracoronary imaging is often deferred in stable patients due to the risk of iatrogenic dissection [1].

The European Society of Cardiology has included cardiac magnetic resonance (CMR) to enhance diagnostic yield in specific ACS subsets, such as SCAD, and it is helpful when differentiating between other etiologies, such as myocarditis or Takotsubo cardiomyopathy [7]. It is typically utilized when there is diagnostic uncertainty; however, a normal CMR cannot rule out SCAD. Cardiac computed tomography angiography (CTA) has been used for diagnosis and to monitor healing; however, there are no routine guidelines yet to monitor SCAD healing [8]. Thus, coronary angiography remains the mainstay of diagnosis for patients with SCAD. PCI carries increased risk in SCAD cases, as there is a potential for the coronary wire to enter the false lumen, worsening the dissection [6]. Managing SCAD in unstable patients remains particularly challenging. Due to its rarity, there is a lack of randomized clinical trials guiding the management of both stable and unstable SCAD patients, leading to variability in treatment approaches among physicians.

Currently, conservative management is preferred for SCAD unless ongoing ischemia or hemodynamic instability necessitates intervention such as with PCI. This patient’s management aligns with current guidelines, which emphasize conservative care and vigilant monitoring [9]. However, most studies on SCAD lesion angiography post conservative therapy are observational and retrospective, and no specific guidelines exist regarding follow-up or the need for repeated coronary angiograms [3]. This underscores the need for further investigation into the long-term effects of SCAD after conservative management and the potential role of additional imaging in follow-up. SCAD generally has a favorable prognosis with appropriate management, though recurrence remains a possibility. Long-term follow-up is crucial, particularly given the risk of recurrence [4,9].

## Conclusions

As the incidence of SCAD has been increasing in recent years, it is important to have a firm understanding of the disease. SCAD typically affects young women without cardiovascular risk factors; however, it should remain part of the differential diagnosis even in patients with traditional risk factors, as seen in our case. While maintaining SCAD in our differential diagnosis, there can be prompt recognition of disease with appropriate imaging with coronary angiography, which is critical for diagnosis, as initial presentations mimic typical ACS. Treatment with either conservative therapy or interventional management should be individualized, as our case demonstrated successful outcomes with conservative management. However, given the chance of recurrence and limited long-term data about SCAD, standardized follow-up protocols need to be established through future research. Practitioners must maintain a high index of suspicion for SCAD in all women presenting with ACS symptoms, regardless of their cardiovascular risk factors, to ensure efficient diagnosis and appropriate treatment.
